# Aminopolycarboxylic Acids-Functionalized Chitosan-Based Composite Cryogels as Valuable Heavy Metal Ions Sorbents: Fixed-Bed Column Studies and Theoretical Analysis

**DOI:** 10.3390/gels8040221

**Published:** 2022-04-05

**Authors:** Maria Valentina Dinu, Ionel Humelnicu, Claudiu Augustin Ghiorghita, Doina Humelnicu

**Affiliations:** 1 “Mihai Dima” Department of Functional Polymers, “Petru Poni” Institute of Macromolecular Chemistry, Grigore Ghica Voda Alley 41A, 700487 Iasi, Romania; claudiu.ghiorghita@icmpp.ro; 2 Faculty of Chemistry, Alexandru Ioan Cuza University of Iasi, Carol I Bd. 11, 700506 Iasi, Romania; ionel@uaic.ro (I.H.); doinah@uaic.ro (D.H.)

**Keywords:** aminopolycarboxylic acids, chelating agents, theoretical analysis, fixed-bed column studies, multicomponent heavy metal ions solution

## Abstract

Over the years, a large number of sorption experiments using the aminopolycarboxylic acid (APCA)-functionalized adsorbents were carried out in batch conditions, but prospective research should also be directed towards column studies to check their industrial/commercial feasibility. In this context, sorption studies of five-component heavy metal ion (HMI) solutions containing Zn^2+^, Pb^2+^, Cd^2+^, Ni^2+^, and Co^2+^ in equimolar concentrations were assessed in fixed-bed columns using some APCA-functionalized chitosan-clinoptilolite (CS-CPL) cryogel sorbents in comparison to unmodified composite materials. The overall sorption tendency of the APCA-functionalized composite sorbents followed the sequence Co^2+^ < Zn^2+^ < Cd^2+^ ≤ Pb^2+^ < Ni^2+^, meaning that Co^2+^ ions had the lowest affinity for the sorbent’s functional groups, whereas the Ni^2+^ ions were strongly and preferentially adsorbed. To get more insights into the application of the composite microbeads into continuous flow set-up, the kinetic data were described by Thomas and Yoon–Nelson models. A maximum theoretical HMI sorption capacity of 145.55 mg/g and a 50% breakthrough time of 121.5 min were estimated for the column containing CS_EDTA_-CPL cryogel sorbents; both values were much higher than those obtained for the column filled with pristine CS-CPL sorbents. In addition, desorption of HMIs from the composite microbeads in dynamic conditions was successfully achieved using 0.1 M HCl aqueous solution. Moreover, a theoretical analysis of APCA structures attached to composite adsorbents and their spatial structures within the complex combinations with transition metals was systematically performed. Starting from the most stable conformer of EDTA, coordinative combinations with HMIs can be obtained with an energy consumption of only 1 kcal/mole, which is enough to shift the spatial structure into a favorable conformation for HMI chelation.

## 1. Introduction

**Figure 1:**
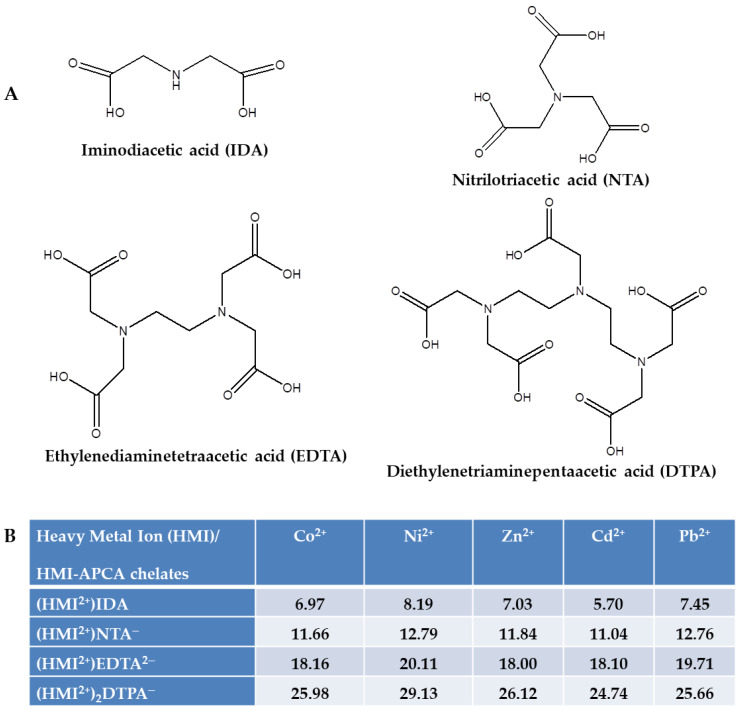
(**A**) Chemical structures of the most important aminopolycarboxylic acids (APCAs) used in heavy metal ions (HMIs) removal/recovery processes; (**B**) pK values for some HMIs-APCAs chelates, Adapted with permission from reference [6], 2013, Elsevier Ltd.

The presence of heavy metal ions (HMIs) in the environment generates huge problems due to their high toxicity and non-biodegradability, and, therefore, their removal by eco-friendly and low-cost sorbents is a “must-do” requirement for manufacturing or processing industrial activities [1,2]. Aminopolycarboxylic acids (APCAs) are well-known as an important group of chelating agents that form stable structures with HMIs [3,4]. They have several carboxylate groups attached to one or more nitrogen atoms capable of strongly binding HMIs. Iminodiacetic acid (IDA), nitrilotriacetic acid (NTA), ethylenediaminetetraacetic acid (EDTA), and diethylenetriaminepentaacetic acid (DTPA) are the most widely used APCAs (Figure 1A): (i) to remove HMIs from aqueous environments, (ii) to prevent the generation of HMIs precipitates, (iii) to impede catalysis mediated by HMIs, or (iv) to control HMIs solubility/availability in the aqueous medium [5]. The broad range of applications of APCAs is attributed to their structural characteristics and unique properties to form stable and water-soluble chelates with HMIs. The stability of the formed chelates is directly connected with the number of the carboxylate groups in the structure of APCAs: the higher their number the higher the stability of the HMIs-APCA chelates, i.e., the pK values (Figure 1B) [6].

Consequently, the surface of a broad range of adsorbent materials has been successfully decorated with APCAs ligand groups, and their adsorption performance and selectivity towards various HMIs has been intensively studied [6,7,8,9,10,11,12,13]. For instance, high adsorption capacities towards Cu^2+^, Co^2+^, Ni^2+^, Pb^2+^, Zn^2+^, and Cd^2+^ ions were reported for acrylonitrile-divinylbenzene copolymers modified by IDA moieties [7,8]. Excellent sorption ability for Cr^3+^ ions of NTA-modified magnetic mesoporous microspheres was also demonstrated [13]. APCAs-modified chitosan (CS) derivatives were also prepared and investigated for removal and recovery of Cu^2+^ [14,15], Ni^2+^ [16,17], Co^2+^ [17], Pb^2+^ [18,19,20,21], or rare earths [22] from simulated aqueous solutions. Bio-derived trifunctional CS−EDTA-β−cyclodextrin sorbents were designed and tested for the simultaneous adsorption of various HMIs (Pb^2+^, Cd^2+^, Hg^2+^, Ni^2+^, and Cu^2+^) and organic pollutants (bisphenol-S, ciprofloxacin, procaine, imipramine, methylene blue, crystal violet, and safranin O) from wastewaters [23,24].

Recently, our group has systematically analyzed the sorption behavior of Cu^2+^ ions from mono-component aqueous solutions onto CS-EDTA cryobeads [15]. A fast kinetic rate, a remarkable reusability, and a sorption capacity of 168 mg Cu^2+^/g were demonstrated for the EDTA functionalized sorbents [15]. The Fe^3+^, Co^2+^, and Cu^2+^ ions sorption in binary and ternary systems by porous EDTA-CS and DTPA-CS composite derivatives was also explored [25]. It was found that the experimental sorption capacity for removal of Fe^3+^ ions from their mixture with Cu^2+^ ions increased with the number of carboxylate moieties attached to the CS support matrix, a sorption capacity of 161.60 mg/g, 189.61 mg/g, and 206.65 mg/g being found for CS, CS-EDTA and CS-DTPA, respectively. Thus, surface functionalization of sorbent materials with APCAs ligand groups has been found to provide them with better adsorptive properties.

So far, the sorption experiments using the APCAs-functionalized adsorbents were mainly carried out in batch conditions, but prospective research should also be directed towards column, pilot-plant, and full-scale studies to check their feasibility at the industrial/commercial level. Moreover, the stability of APCAs ligands attached to adsorbent materials should be regularly supervised during the HMIs sorption to ensure that the leaching of APCA moieties is not occurring in the treated wastewaters. Herein, as the real-life aqueous environments are composed of more complex HMIs mixtures, sorption studies towards five-component metal ion solutions were assessed in fixed-bed columns. Thereafter, to examine the feasibility of APCA-functionalized CS-clinoptilolite (CPL) composite cryogel sorbents for treatment of HMI-containing wastewaters, a theoretical analysis of EDTA and DTPA structures in complex combinations with transition metal ions was also performed.

## 2. Results and Discussion

**Figure 2:**
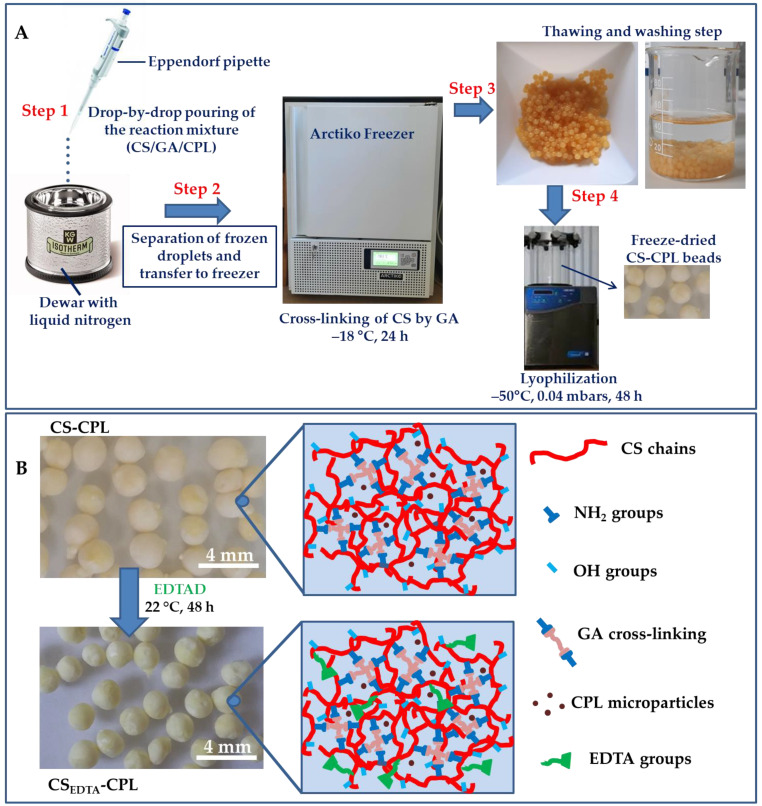
(**A**) A schematic procedure of the preparation of CS-CPL sorbents. (**B**) Optical pictures showing freeze-dried CS-CPL microbeads before and after interaction with 4,4′–ethylenebis(2,6– morpholinedione) (EDTAD) and schematic illustration of –NH_2_, -OH, and EDTA functional groups available within CS-CPL and CS_EDTA_-CPL composite sorbents available for interaction with HMIs.

In this work, chemically cross-linked CS-CPL composite cryogel sorbents by glutharaldehyde (GA) with a high porosity and large pore sizes were prepared by a cryogelation process, according to a procedure previously reported [26]. Briefly, CS-CPL composite cryogels were formed as beads by pouring a homogeneous dispersion consisting of CS, CPL, and GA into liquid nitrogen using an Eppendorf pipette (Step 1, Figure 2A). The frozen droplets were then separated from liquid nitrogen and immediately transferred in an Arctiko Freezer at −18 °C to ensure the complete cross-linking of CS by GA (Step 2, Figure 2A). After 24 h, the CS-CPL beads were thawed at room temperature and washed with MilliQ water to remove unreacted compounds (Step 3, Figure 2A). Thereafter, the CS-CPL composite beads were freeze-dried in a LABCONCO FreeZone apparatus for 48 h, at –50 °C and 0.04 mbar (Step 4, Figure 2A). The CS-CPL sorbents were further modified with EDTA moieties to enhance the sorption performance of the pristine composites. The introduction of EDTA ligand moieties onto CS-CPL sorbents was achieved by reaction with 4,4′–ethylenebis(2,6– morpholinedione) (EDTAD) in a acetic acid-methanol (1:1 *v/v*) mixture at 22 °C for 48 h (Figure 2B). The energy dispersive X-ray analysis (EDX) on the surface of CS-CPL and CS_EDTA_-CPL microbeads (Table 1) proved the presence of C, N, O, Al, Si, Na, K, and Ca elements from CS and CPL, revealing the good stability of CPL microparticles within the CS matrix during the functionalization step. In addition, the higher atomic ratio percentages found for N in the case of CS_EDTA_-CPL microbeads is a further indication of the successful modification of CS-CPL sorbents with EDTA functional groups. The evaluation of porosity, pore sizes, and Brunauer–Emmett–Teller (BET_H2O_) surface area of composite sorbents (Table 2) pointed out the presence of a macroporous structure for both sorbents, which could provide a fast access of HMIs to a large number of chelating sites.

### 2.1. Experimental Data on HMIs Sorption in Fixed-Bed Column

**Figure 3:**
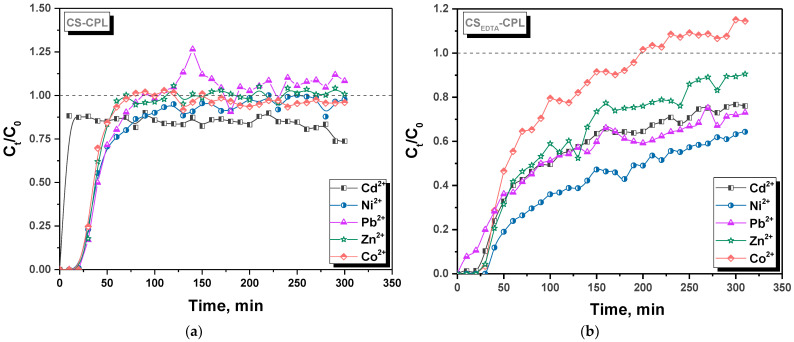
Experimental breakthrough curves for individual HMIs sorption from simulated multicomponent solutions by CS-CPL (**a**) and CS_EDTA_-CPL (**b**) microbeads in fixed-bed column experiments.

Analysis of sorbents efficiency in fixed-bed column sorption experiments is performed with the consideration of their upscaling towards real wastewater treatment applications [28,29,30,31]. The performance of fixed-bed columns is described by the breakthrough curves, which are representations of normalized effluent concentration as a function of flow time or bed volume. In this work, the performance of CS-CPL and CS_EDTA_-CPL microbeads in continuous flow set-ups was investigated, under competitive conditions, using a simulated aqueous solution containing Zn^2+^, Pb^2+^, Cd^2+^, Ni^2+^, and Co^2+^ in equimolar concentrations (C = 0.5 mmol/L). The breakthrough curves for each individual metal ion for the CS-CPL and CS_EDTA_-CPL supports are depicted in Figure 3.

For both types of microbeads, a certain degree of chromatographic effect was observed, due to the competition of solute species for the available functional groups. The column filled with CS-CPL microbeads showed much steeper breakthrough profiles for all tested HMIs (especially for Cd^2+^) compared to the one containing CS_EDTA_-CPL microbeads. This is because the sorbent’s available active sites saturate more rapidly, i.e., it has a lower sorption capacity. For the column containing CS_EDTA_-CPL microbeads, a clear separation of the breakthrough profiles was observed, as a result of the sorbent’s different affinity for the HMIs. Thus, the interaction order followed the sequence Co^2+^ < Zn^2+^ < Cd^2+^ ≤ Pb^2+^ < Ni^2+^, meaning that Co^2+^ had the lowest affinity for the sorbent’s functional groups, whereas between Ni^2+^ and the CS_EDTA_-CPL microbeads, the strongest interactions were established. The obtained sequence for CS_EDTA_-CPL microbeads is well correlated to the sequence of the HMIs-EDTA chelates pK values listed in Figure 1B.

**Figure 4:**
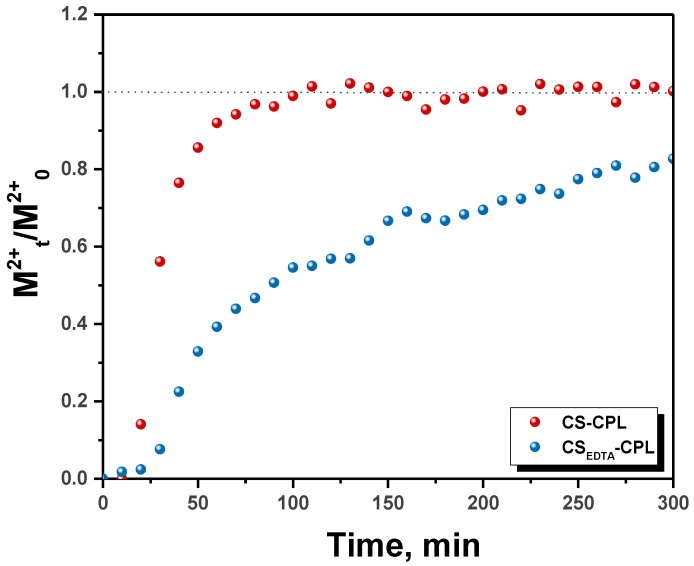
The CS-CPL and CS_EDTA_-CPL columns saturation with HMIs as a function of time.

To be effective in column set-ups, a material should possess high adsorption capacity as well as fast sorption kinetics for the target solutes [32]. In order to gain more insight into the columns performance, the breakthrough curves for the two tested systems were represented for the sum of all HMIs in the solution (Figure 4).

As seen in Figure 4, the column with CS-CPL microbeads was exhausted quickly, after approximately 100 min, whereas for the one with CS_EDTA_-CPL microbeads only approximately 80% saturation was found after 300 min. This shows that CS_EDTA_-CPL microbeads have a much higher sorption capacity for HMIs, as already presented in our recent work [15,25] in batch sorption experiments.

In order to get more insights into the application of CS-CPL and CS_EDTA_-CPL beads in continuous flow set-up, the kinetic data obtained in the fixed-bed experiments were fitted with the Thomas and Yoon–Nelson models, and the fitting results are listed in Table 3. The Thomas model (Equation (1)) was derived from the Langmuir adsorption isotherm and the second-order reaction kinetics, under the assumption of zero longitudinal dispersion in the fixed bed [33]:

(1) $$\;\frac{C_{t}}{C_{0}}=\frac{1}{1+\exp\left[\left(\frac{k_{Th}q_{0}m}{Q}\right)-k_{Th}C_{0}t\right]}\;,$$

where *C_t_* (mg/L) and *C_0_* (mg/L) are the concentration of HMIs in effluent and influent, respectively, *k_Th_* is the Thomas rate constant (L/mg∙min), *q_0_* (mg/g) is the maximum sorption capacity, *m* (mg) is the mass of adsorbent, and *Q* (mL/min) is the flow rate.

The Yoon–Nelson model (Equation (2)) is based on the assumption that the expected sorption decrease rate is related to the breakthrough of the adsorbent [34]:

(2) $$\frac{C_{t}}{C_{0}}=\frac{1}{1+\exp\left(k_{YN}\left(\tau -t\right)\right)}\;,$$

where *k_YN_* (mL/min·mg) represents the Yoon–Nelson reaction constant, and *τ* (min) is the time at which 50% breakthrough is achieved.

The parameter q_0_ in the Thomas model provides an estimate for the maximum sorption capacity of a sorbent in dynamic conditions, whereas τ in the Yoon–Nelson model gives the time for 50% breakthrough. Thus, for the column containing CS_EDTA_-CPL microbeads, a maximum HMI sorption capacity of 145.55 mg/g and a 50% breakthrough time of 121.5 min were estimated, with both being much higher than for the column containing CS-CPL microbeads. In addition, the kinetic data obtained in the fixed-bed experiments for CS_EDTA_-CPL cryogel sorbents towards each HMI were further analyzed by the Thomas and Yoon–Nelson models. The maximum theoretical sorption capacities of the CS_EDTA_-CPL composite sorbent for Co^2+^, Zn^2+^, Cd^2+^, Pb^2+^, and Ni^2+^ ions were found to be 79.57, 130.03, 158.72, 166.78, and 249.53 mg/g, respectively (Table S1). A 50% breakthrough time of 66.43, 108.56, 132.52, 139.24, and 208.34 min was assessed for Co^2+^, Zn^2+^, Cd^2+^, Pb^2+^, and Ni^2+^ ions, respectively (Table S1). The efficiency of the composite sorbents prepared in this study was compared with that of other sorbents reported in the literature (Table S2). Albeit all sorbents included in Table S2 can be exploited for the removal of HMIs from multicomponent mixtures, our CS_EDTA_-CPL sorbent exhibited the highest sorption capacity. Overall, it is clearly demonstrated that the modification of CS based structures with APCA moieties is a powerful strategy to significantly improve their HMI chelation properties, the obtained materials being highly suitable for water purification in continuous flow conditions.

To ascertain the functional groups involvement in the sorption of HMIs by CS-CPL and CS_EDTA_-CPL microbeads, FTIR spectra of the microbeads before and after the contact with HMI multicomponent solution were recorded (Figures S1 and S2, Supporting Information). Thus, the FTIR spectrum of pristine CS-CPL microbeads presents characteristic bands for stretching vibrations of -OH and -NH groups (at 3435 cm^−1^), stretching vibrations of -CH and CH_2_ groups (at 2926 cm^−1^ and 2878 cm^−1^), C=O stretching (at 1651 cm^−1^), -NH_2_ bending (at 1564 cm^−1^), CH_2_ bending (at 1414 cm^−1^), CH_2_ deformations (at 1383 cm^−1^), C-O-C bridges deformation vibrations (at 1151 cm^−1^), and C-O stretching (1082 cm^−1^ and 1036 cm^−1^) that are attributed to the CS matrix [35,36]. The presence of CPL particles within the microbeads is supported by the characteristic bands at 463 cm^−1^, 609 cm^−1^, and 795 cm^−1^ [37]. However, the absence of the band characteristic to -NH_2_ bending vibrations, as well as the very broad bands at 1641 cm^−1^ and 1383 cm^−1^ (attributed to asymmetric and symmetric stretching vibrations of -COO^-^ groups) in the spectrum of CS_EDTA_-CPL microbeads support the successful functionalization with EDTA moieties [15]. The sorption of HMIs by both microbeads led to several spectral changes. Thus, the disappearance of the -NH_2_ group bending vibrations from the spectrum of CS-CPL microbeads, after the interaction with the HMIs supports the involvement of these groups in the sorption process. In the spectrum of CS_EDTA_-CPL microbeads after interaction with HMIs, the band at 1589 cm^−1^ (blue-shifted as in the spectrum of corresponding pristine sample) indicates the involvement of carboxyl groups in the complexation of HMIs.

**Figure 5:**
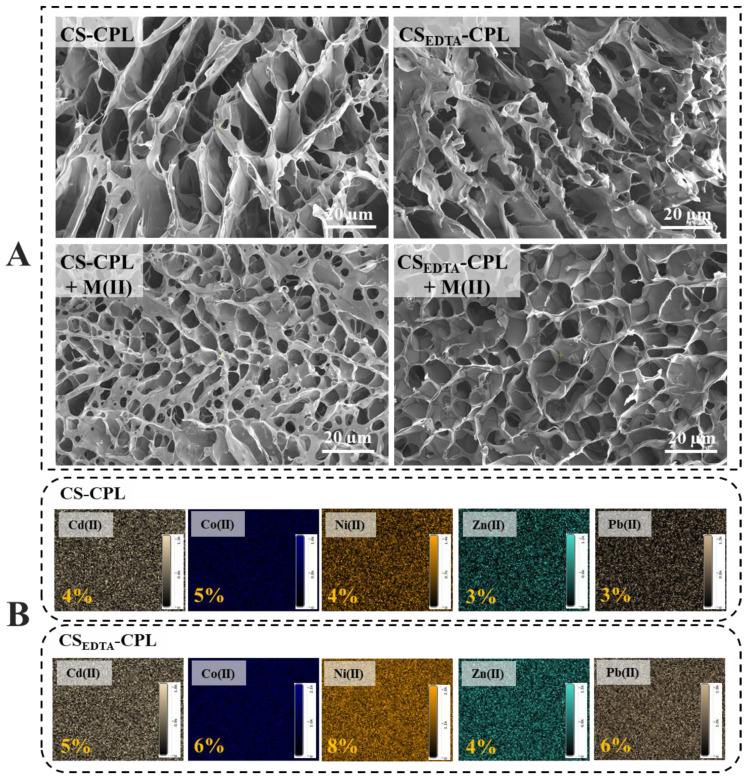
(**A**) SEM micrographs of CS-CPL and CS_EDTA_-CPL microbeads before and after interaction with the multicomponent HMIs solution; (**B**) EDX mapping and elemental analysis of CS-CPL and CS_EDTA_-CPL microbeads surface after interaction with the HMI solution.

The internal structure of CS-CPL and CS_EDTA_-CPL microbeads before and after interaction with the multicomponent HMI solution was analyzed by SEM (Figure 5A). A heterogeneous porous morphology with interconnected pores could be observed for all samples. The mean pore sizes of the CS-CPL and CS_EDTA_-CPL before HMI sorption were approximately 21.49 ± 5.27 μm and 30.16 ± 6.18 μm, whereas after M(II) sorption their diameters drastically decreased to approximately 5.65 ± 1.42 μm and 5.87 ± 1.26 μm, respectively, which indicates a strong interaction between HMIs and functional groups of the support matrix. The EDX mapping of CS-CPL and CS_EDTA_-CPL microbeads surface after interaction with the HMI solution (Figure 5B) shows the uniform distribution of each HMI on the sorbent surface. The elemental analysis of CS_EDTA_-CPL after M(II) sorption further sustains the high affinity of this sorbent for Ni^2+^ ions (8% atomic ratio—Figure 5B).

**Figure 6:**
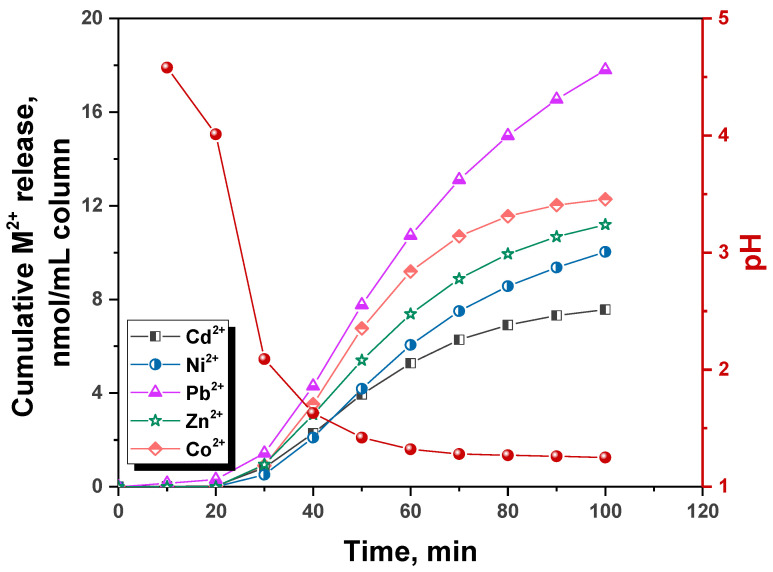
Cumulative M^2+^ release (nmol/mL column) in dynamic conditions and effluent pH during desorption of HMIs with 0.1M HCl from the CS_EDTA_-CPL microbeads.

The desorption of HMIs from the CS_EDTA_-CPL microbeads was also investigated in dynamic conditions, using 0.1 M HCl aqueous solution as eluent. In Figure 6, the cumulative release amounts of all HMIs (in nmol/mL column), in tandem with the effluent pH evolution, as a function of elution time is depicted.

In acidic media, the amino and carboxyl groups from CS backbone and EDTA moieties, respectively, are protonated, the HMIs being thus desorbed from the sorbent. As it is seen, the pH of collected effluent quickly decreased in the first 30 min of the desorption experiment down to around pH 2, afterwards swiftly evolving towards an equilibrium value. Simultaneously, the release of HMIs was low in the first 30 min of the desorption experiment, but quickly increased afterwards. The cumulative HMI amounts determined after 100 min of running time were 17.8 nmoles of Pb^2+^, 7.56 nmoles of Cd^2+^, 12.28 nmoles of Co^2+^, 10.03 nmoles of Ni^2+^, and 11.19 nmoles of Zn^2+^ (calculated with respect to mL column). Overall, the dynamic desorption experiment highlights the possibility that the microbeads investigated in this work can be cleaned of the sorbed HMIs in continuous flow set-ups, with potential for regeneration and reuse in successive sorption/desorption cycles without the need to remove the materials from the column.

**Figure 7:**
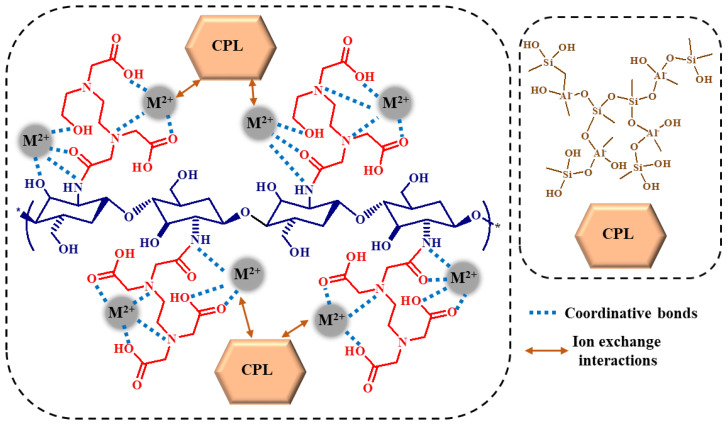
Possible mechanism for the sorption of HMIs onto the CS_EDTA_-CPL cryogel sorbents.

A schematic representation of the possible HMI sorption mechanism for CS_EDTA_-CPL cryogel sorbent is depicted in Figure 7. The coordinative bonds between the functional groups of modified CS network and HMIs and the ion exchange between the Na^+^, K^+^, and Ca^2+^ from CPL and Co^2+^, Zn^2+^, Cd^2+^, Pb^2+^, and Ni^2+^ ions are the most probable processes according to experimental and characterization results.

### 2.2. Theoretical Analysis of EDTA and DTPA Structures Attached to CS-CPL Composite Adsorbents and Their Spatial Structures within the Complex Combinations with Transition Metals

Using the Gaussian and hybrid functional B3LYP program package [38,39,40] together with the 6–31 g orbital set, the structures of EDTA and DTPA were investigated to identify the most energetically stable conformer, the interaction with a CS unit, as well as their spatial structures within the complex combinations with transition metal ions. The theoretical investigations were performed in vacuum considering a single structural unit, without solvent influences or symmetry constraints. In addition, the polarization functions corresponding to elements that are in the periodic system at least in the second period were also considered. Under these conditions, five structures were identified for EDTA whose relative energy (kcal/mole), spatial distribution, and electric dipole moment are included in Table 4.

The conformer, EDTA-1, has two hydrogen bonds in its structure, between the carboxyl groups found at the same N atom, which contribute to the stabilization of the spatial distribution, being thus the most energetically stable structure with a symmetry characterized by zero dipole moment (Table 4). The next conformer of the EDTA structure (EDTA-2) has a hydrogen bond between the hydroxyl group and the N atom to which the acidic moiety is bonded. The conformer of EDTA-2 displays a ‘zig-zag’ structure, which explains the high value of the dipole moment of over 6 Debye. In the structure of EDTA-3 and of the following conformers, no hydrogen bonding interaction contributing to the energy stabilization of the conformers is observed. In terms of energy difference from the previous conformer, the difference is not very large, less than one kcal/mole, but one can observe the difference in spatial distribution reflected by the dipole moment value, which is approximately 4 Debye. EDTA-4 is characterized by the existence of a symmetry plane perpendicular to the main chain, but also by the rotation of the carboxyl groups symmetric to the C-C bond within the substituent. The last conformer considered, EDTA-5, is characterized by a perpendicularity of the planes in which the substituent carboxyl structures are located to the same N atom.

**Figure 8:**
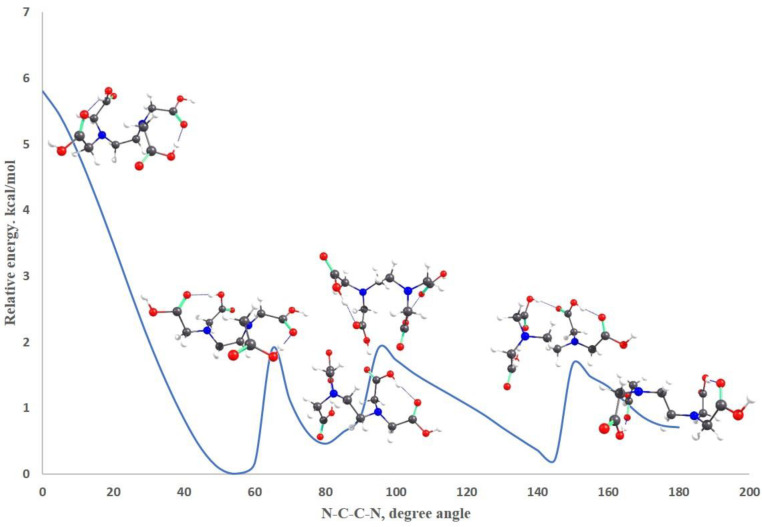
The variation of the relative energy considering the values of the dihedral angle N-C-C-N.

It should be pointed out that the EDTA structure characterized by a high energetic stability, EDTA-1, does not have a spatial distribution that favors the formation of complex combinations with HMIs. However, to ensure this, the spatial structure of EDTA-1 conformer has to undergo a rotation around the central C-C bond. A graphical representation of the relative energy variation registered by modifying the N-C-C-N dihedral angle from 180 degrees, as it is approximately in EDTA-1, to zero degrees is illustrated in Figure 8. It was found that a complete rotation requires a relative energy of about 6 kcal/mole (Figure 8). The changes in spatial structure during rotation shows the formation of a molecular framework favorable to the generation of coordinative structures with metal ions even from an angle of 145 degrees, which requires a rotational energy of 1 kcal/mole. Similar energy barriers, around 1 kcal/mole, also characterize the other hypothetic structures favorable to the formation of complex combinations with transition metal ions. As the dihedral angle decreases towards zero, the rotational energy increases to 6 kcal/mole. It can be concluded that starting from the most stable conformer of EDTA, coordinative combinations with metal ions can be obtained with an energy consumption of only 1 kcal/mole, which is enough to shift the spatial structure into a favorable conformation for metal ion chelation.

Compared to EDTA, the structure of DTPA is more complex, having an additional aminocarboxylic fragment inserted into the main chain (Figure 1A). The conformer of DTPA with the lowest energy has been also determined by applying similar calculations as in the case of EDTA structure. In Table 5, the top five conformer structures of DTPA have been selected with respect to their relative energy.

The most energetically stable DTPA structure contains four interactions of hydrogen-bonding type (Table 5): (i) two between the hydrogen atom from hydroxyl groups that are part of the carboxyl residues attached to marginal N atoms of the main chain and the ketone O atoms of the other group, and (ii) two between the hydroxyl groups of the other acid functions of the marginal N atom. The first set of interactions is exerted at a distance of approximately 1.78 Å, whereas the second one is at approximately 2.0 Å. Analyzing the structures presented in Table 5, it can be seen that these intra-molecular interactions through hydrogen bonds characterize all the selected conformers. For EDTA, the most energetically stable conformer had an electric dipole moment of zero, whereas in the case of DTPA, we obtained a value higher than 7 Debye, which is larger than that of the other conformer structures listed in Table 5. The second conformer structure, DTPA-2, contains five hydrogen bonds; the additional one corresponds to the interaction between the hydroxyl group of the acid function located at the N atom in the center of the main chain and the corresponding N atom and is on the opposite side of the plane containing the main chain atoms. This spatial distribution of the molecule led to a decrease in the dipole moment value to approximately 5.45 Debye. In the DTPA-3 conformer, both carboxyl groups that form a hydrogen bond with the N atom to which they bind are on opposite sides of the molecular plane, leading to a further decrease in the dipole moment to 3.36 Debye due to the increased symmetry of the molecular system. If a hydrogen-bonding interaction is formed between two carboxyl groups attached to a marginal and central N atom on the same side of the molecular plane, the DTPA-4 conformer structure is obtained. The new interaction is established at a distance of approximately 1.8 Å between the H atom of the hydroxyl group of the acid group bound to the marginal N atom and the O in the hydroxyl group located at the central N atom, which is also involved in a similar interaction with that N atom. In the structure of the last conformer considered here, DTPA-5, there are five hydrogen bonds; one of the second types is on the opposite side of the molecular plane from the other two of the same type made at the others marginal and central N atoms. This spatial distribution of atoms can be achieved by reorienting the interaction from the previous conformer between the OH groups of two acid groups on the same side of the main chain plane towards the marginal N atom to which the functional group is bonded.

**Figure 9:**
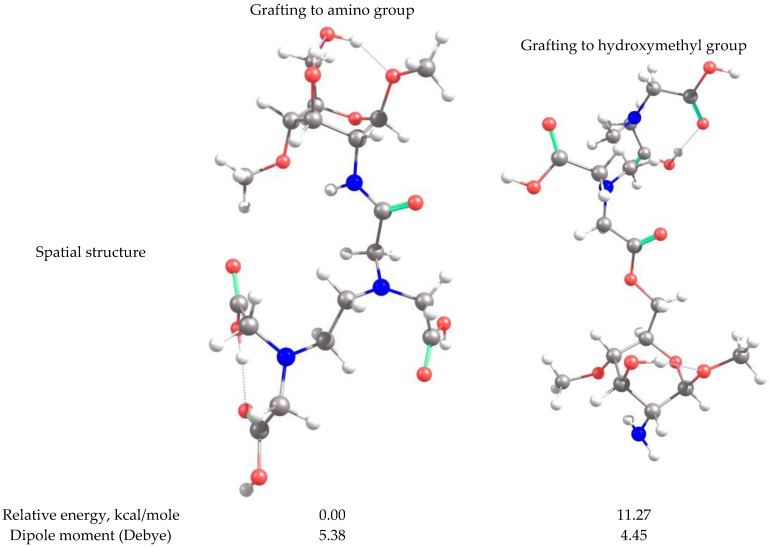
Spatial structures of EDTA grafted onto the CS unit and the corresponding values of the relative energy and dipole moment.

Because the structures of APCAs in the HMI sorption studies are not self-standing, but grafted on CS-CPL composite adsorbents, theoretical investigations were also carried out on the spatial conformation of these structures considering that grafting of APCAs can be accomplished either to the O atom of the hydroxymethyl group or to the amino N atom of the CS backbone. Considering the same functional analysis and set of basic orbitals, spatial structures of CS_EDTA_-CPL units were obtained for each grafting modality (Figure 9). It should be noted that only one structural unit was used from the CS chain, and methyl structures were linked to the valences of the marginal atoms. The structure obtained by grafting EDTA to the amino group is energetically more stable than the one resulting from attaching the EDTA residue to the hydroxymethyl group in the CS chain (Figure 9). It should be noted that between the carboxyl groups attached to the same N atom in EDTA, there is an interaction by hydrogen bonding. Analyzing the obtained structures, it can be concluded that in the case of complexation with transition metal ions, coordinative bonds can be formed between three carboxyl groups and metal ions, and also there is a possibility of formation of two dative bonds with N atoms in the EDTA structure.

**Figure 10:**
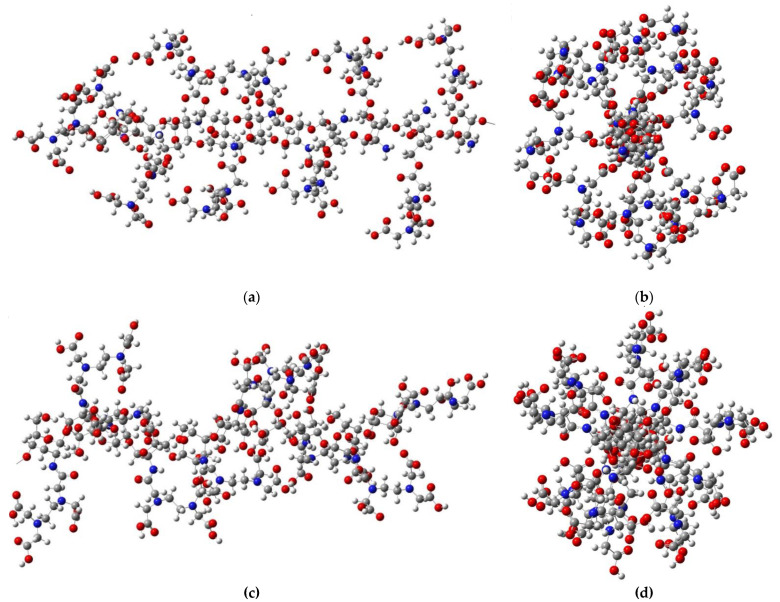
Spatial representation of a structure consisting of ten CS units with EDTA grafted to the hydroxymethyl groups: (**a**) along the chain; (**b**) perpendicular to the chain. Spatial representation of a structure consisting of ten CS units with EDTA grafted to the N atom of the amino groups: (**c**) along the chain; (**d**) perpendicular to the chain.

If the EDTA structures were grafted to each hydroxymethyl functional group within the CS units, the chain of ten structural units would be spatially distributed as shown in Figure 10a. However, from the perpendicular representation on the chain, it can be observed that the EDTA structures are distributed at a certain angle one to another, so that spatial constraints are minimal, resulting in a circular structure around the CS chain (Figure 10b). This distribution can also be associated with the helical spatial structure of the CS itself. Similar structures were also obtained when EDTA was grafted to the amino groups in the CS unit. The distribution of EDTA structures around the CS chain is slightly different from the previous case due to steric hindrances between the grafted structures (Figure 10c,d).

**Figure 11:**
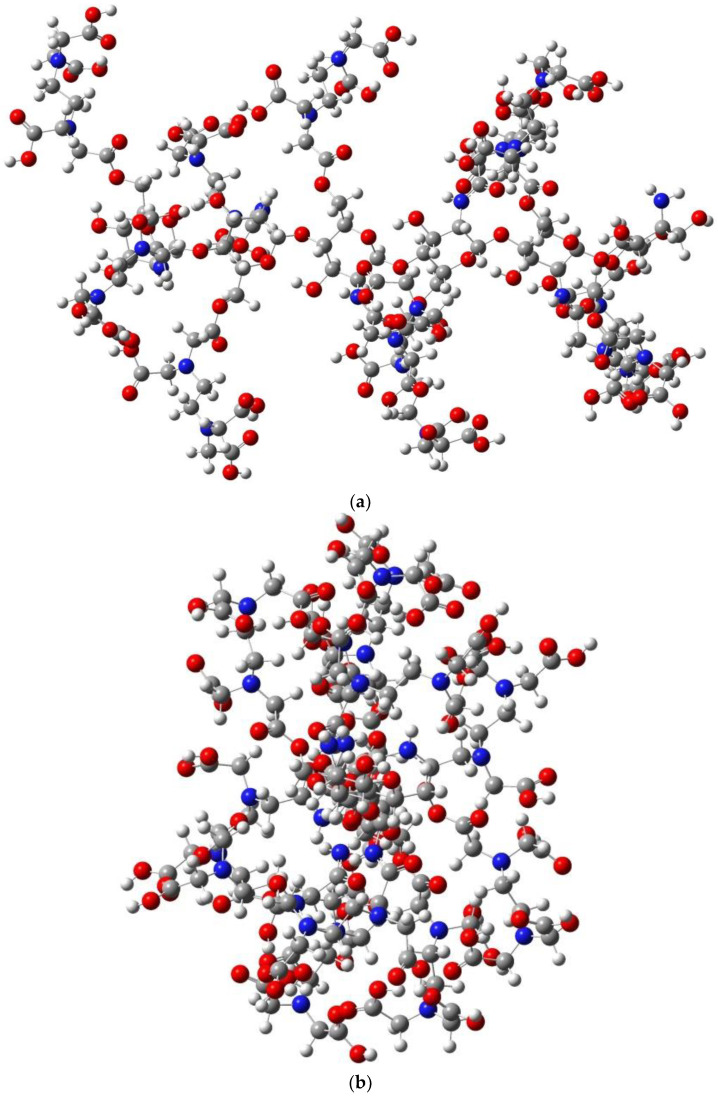
Spatial representation of a structure consisting of six CS units with EDTA grafted alternately to the N atom of the amino groups and the O atom of the hydroxymethyl groups: (**a**) along the chain; (**b**) perpendicular to the chain.

If EDTA grafting to the CS units is done alternately, with one unit grafted to the hydroxymethyl groups and the next to the amino groups, the spatial structure of a chain consisting of six CS units is shown in Figure 11. It can be seen the arrangement of EDTA structures on both sides and the rotation of the grafted structures around the main chain due to steric hindrances and to the helical structure of the CS chain.

### 2.3. Theoretical Analysis on the Electronic Transitions of EDTA and DTPA Structures

**Figure 12:**
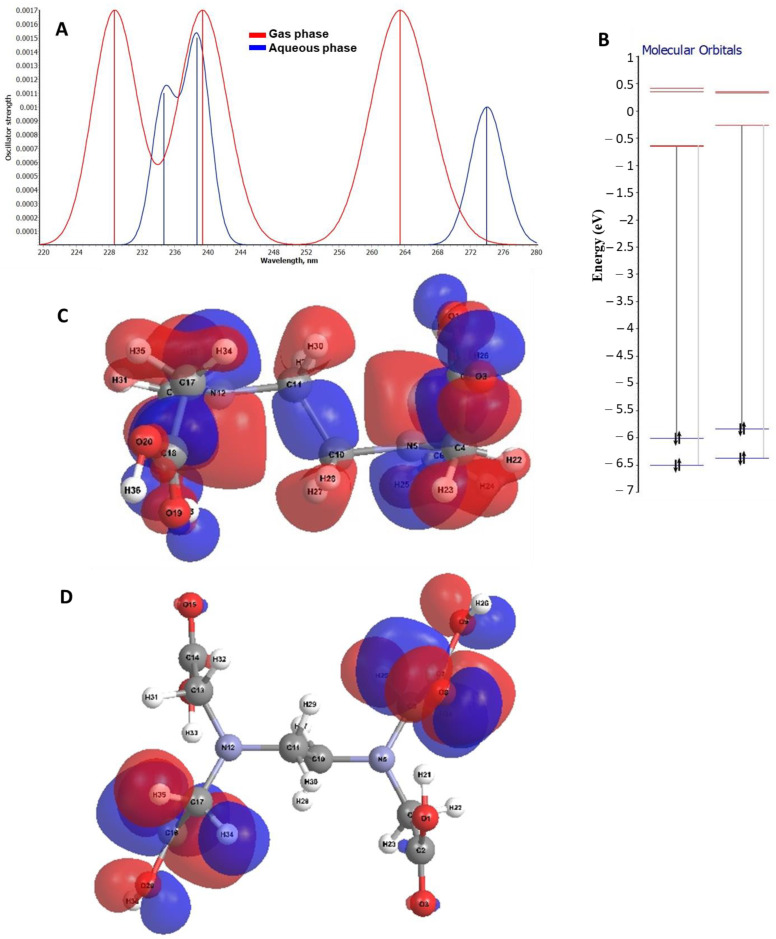
(**A**) Theoretical electronic spectrum for the most energetically stable EDTA conformer corresponding to both gas and aqueous phase. (**B**) Fragment from the energy diagram illustrating electronic transitions between boundary levels for EDTA structure: (a) gas phase; (b) aqueous phase. The spatial representation of the boundary orbitals for EDTA structure in gaseous phase (**C**) HOMO and (**D**) LUMO.

Theoretical investigations were also carried out on the electronic transitions in the EDTA and DTPA molecular structures. Theoretical electronic spectra were simulated both in the gas phase and in water. Thus, for the most stable EDTA structure, the spectral simulations were obtained using the TD-DFT procedure (Figure 12A). A shift of the electronic transitions towards lower wavelengths in solution than in the gas phase was observed. For example, HOMO → LUMO transitions are found in gas at 274 nm, whereas in solution they are found at 263.5 nm. This is due to the interaction of the studied structure with the solvent, which led to an increase in the energy of the LUMO orbital and thus the transition energy at this level. This aspect is also evident in the energetic diagram plotted to illustrate the electronic transitions between boundary levels (Figure 12B). Analyzing the composition of the boundary orbitals, it can be seen that the spectral transitions are determined by the jumping/dropping of electrons from the *pz* orbital located at N atoms to the *px* orbital of C atoms. This can also be seen from the spatial representation of the molecular boundary orbitals in Figure 12C,D.

**Figure 13:**
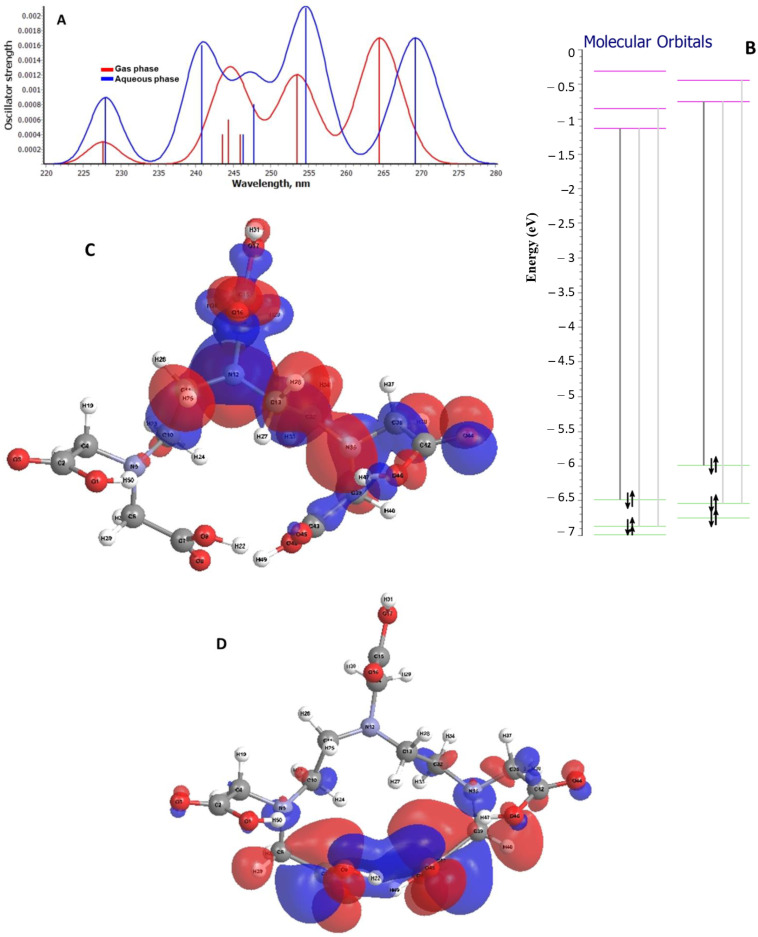
(**A**) Simulation of the electronic spectra for the DTPA structure in both the gas and aqueous phase. (**B**) Fragment from the energy diagram of the molecular orbitals of the DTPA structure illustrating the first electronic transition: (a) gas phase; (b) aqueous phase. The spatial representation of boundary orbitals for DTPA structure in gaseous phase (**C**) HOMO and (D) LUMO.

Regarding the study of electronic transitions within the DTPA molecule, it can be seen from Figure 13A that the HOMO → LUMO transition in the aqueous state is at a lower energy than that in the gas phase, the energy difference being almost 5 nm. In the aqueous phase it can be seen from the energy diagram (Figure 13B) that, following interactions with water molecules, the energy corresponding to the first vacant molecular level is lower than that in the gas phase, which also explains the lower energy of the electronic transition between the boundary levels in solution versus gas. The structure of the DTPA boundary orbitals is illustrated in Figure 13C,D. A transfer of electrons from the atomic orbitals of the atoms inside the chain to the orbitals of the marginal atoms involved in the formation of the hydrogen bond interaction can be observed (Figure 13C,D). Thus, the formation of hydrogen bonds is favored by the interaction with water molecules.

**Figure 14:**
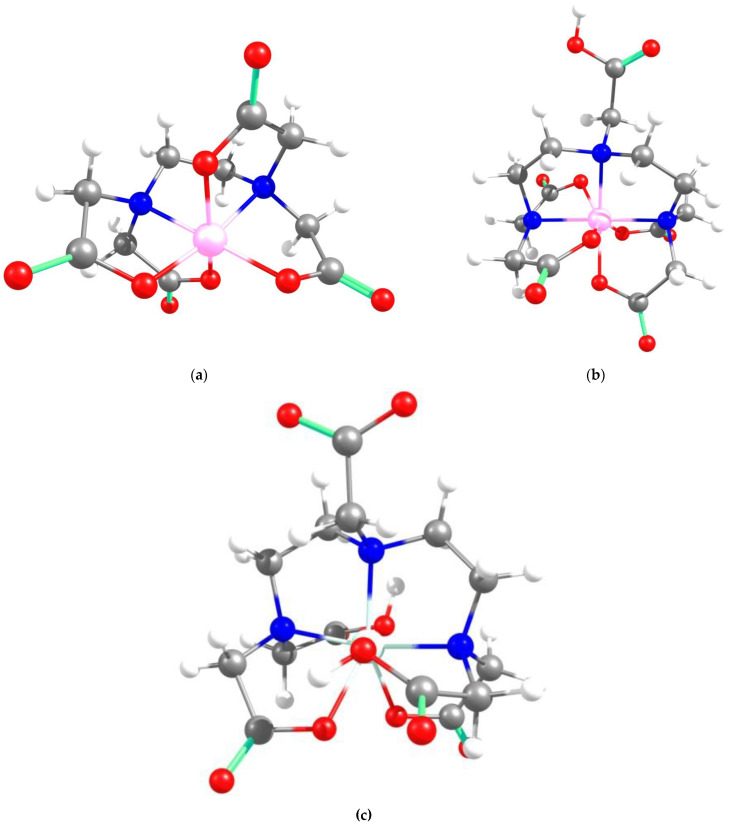
Proposals for the spatial representation of EDTA (**a**) and DTPA (**b**) structures investigated in coordination with transition metal ions. (**c**) The spatial structure for CuC14N3H19O10 with two dative bonds from two carboxyl groups.

Concerning the complexation of EDTA and DTPA structures with metal ions, for the gaseous state, without taking into account that the ligand is grafted onto a CS structure, the spatial distributions of the investigated structures illustrated in Figure 14a,b were proposed. The metal ions form dative bonds with the N atom and coordinative bonds with the O atoms from the carboxyl groups at the end of the chain in the case of the DTPA structure. Depending on solution pH, some carboxyl groups may form dative bonds with the coordinated metal ions, as illustrated in Figure 14c, where a structure with DTPA as ligand and Cu as transition metal ion is represented.

## 3. Conclusions

The sorption performances in fixed-bed columns of APCA-functionalized chitosan-based composite cryogels, as microbeads, towards five-component HMI solutions containing Zn^2+^, Pb^2+^, Cd^2+^, Ni^2+^, and Co^2+^ was thoroughly investigated in this study in comparison to those of unmodified composite sorbents. The column filled with unmodified microbeads showed much steeper breakthrough profiles for all tested HMIs compared to the one containing EDTA-functionalized microbeads. This behavior was associated with the rapid saturation of composite sorbent’s available active sites. For the column containing CS_EDTA_-CPL microbeads, a clear separation of the breakthrough profiles was observed; the interaction order followed the sequence Co^2+^ < Zn^2+^ < Cd^2+^ ≤ Pb^2+^ < Ni^2+^, indicating that Ni^2+^ ions showed the highest affinity for the sorbent’s functional groups. The obtained sequence for CS_EDTA_-CPL microbeads has been well correlated to the sequence of the HMIs-EDTA chelates pK values.

In order to get information about the maximum sorption capacity of a sorbent in dynamic conditions and the time for 50% breakthrough, the kinetic data obtained in the fixed-bed experiments were fitted by Thomas and Yoon–Nelson models. Thus, for the column containing CS_EDTA_-CPL microbeads, a maximum HMIs sorption capacity of 145.55 mg/g and a 50% breakthrough time of 121.5 min were estimated, with both being much higher than for the column containing unmodified CS-CPL microbeads. In addition, the dynamic desorption experiment highlights the possibility that the microbeads investigated in this work can be cleaned of the sorbed HMIs in continuous flow set-ups, with potential for regeneration and reuse in successive sorption/desorption cycles without the need to remove the materials from the column.

Moreover, using the Gaussian and hybrid functional B3LYP program package together with the 6–31 g orbital set, the structures of EDTA and DTPA were investigated to identify the most energetically stable conformer, the interaction with a CS unit as well as their spatial structures within the complex combinations with transition metal ions. It was concluded that starting from the most stable conformer of EDTA, coordinative combinations with metal ions can be obtained with an energy consumption of only 1 kcal/mole, which is enough to shift the spatial structure into a favorable conformation for metal ion chelation. Theoretical investigations were also carried out on electronic transitions in the EDTA and DTPA molecular structures: their electronic spectra were simulated both in the gas phase and in water. Thus, for the most stable EDTA structure, a shift of the electronic transitions towards lower wavelengths in solution than in the gas phase was observed. For example, HOMO→LUMO transitions were found in gas at 274 nm, whereas in solution they were found at 263.5 nm. This is due to the interaction of the studied structure with the solvent, which led to an increase in the energy of the LUMO orbital and thus the transition energy at this level. Concerning the complexation of EDTA and DTPA structures with metal ions, for the gaseous state, it was shown that the metal ions can form dative bonds with the N atom and coordinative bonds with the O atoms from the carboxyl groups at the end of the chain. Overall, it was clearly demonstrated that the modification of CS based structures with APCA moieties is a powerful strategy to significantly improve their HMIs chelation properties, the obtained materials being highly suitable for water purification in continuous flow conditions.

## 4. Materials and Methods

### 4.1. Materials

CS-CPL and CS_EDTA_-CPL composites, as microbeads, were used as sorbents for removal of Zn^2+^, Pb^2+^, Cd^2+^, Ni^2+^, and Co^2+^ ions from multicomponent aqueous solution. The preparation of CS-CPL and CS_EDTA_-CPL composites has been previously published [15,25]. Briefly, CS-CPL composites chemically cross-linked by glutaraldehyde (GA), as microbeads, were prepared by a cryogenic process in three stages: (i) freezing of synthesis mixture as droplets at—196 °C using liquid nitrogen; (ii) storage in the frozen state at −18 °C for 24 h to complete CS cross-linking by GA; (iii) thawing at room temperature for 1 h and washing with MilliQ water to remove the unreacted compound; and (iv) lyophilization for 48 h, at –50 °C and 0.04 mbar. Next, a part of the CS-CPL composites was functionalized with EDTA moieties, according to a protocol already reported [25]. Pb(NO_3_)_2_ (≥99%); Zn(NO_3_)_2_·6H_2_O (≥99%); Ni(NO_3_)_2_·6H_2_O (≥97%); Cd(NO_3_)_2_·4H_2_O (≥99%); Co(NO_3_)_2_·6H_2_O (≥99%) were purchased from Fluka (Sigma-Aldrich, Steinheim, Germany) and were used as received.

### 4.2. Methods

#### 4.2.1. SEM and EDX Analysis

Scanning electron microscopy (SEM) studies were performed using a Verios G4 UC (Thermo Scientific, Brno, Czech Republic) device equipped with energy dispersive X-ray (EDX) analyzer (Octane Elect Super SDD detector, Mahwah, NJ, USA). The CS-CPL and CS_EDTA_-CPL microbeads were coated with a 10 nm platinum layer using a Leica EM ACE200 Sputter to provide electrical conductivity and to prevent charge build-up during exposure to the electron beam. SEM analysis was carried out in high vacuum mode using a secondary electron detector (Everhart-Thornley Detector, Thermo Scientific, Brno, Czech Republic) at an accelerating voltage of 5 kV.

#### 4.2.2. FTIR Analysis

FTIR spectra of composite microbeads, before and after HMI sorption, were recorded by a Bruker Vertex FT-IR spectrometer (Bruker, Ettlingen, Germany), at a resolution of 2 cm^−1^, in the range 4000–400 cm^−1^, by KBr pellet technique.

#### 4.2.3. Porosity Evaluation

The porosity (P, %) of CS-CPL and CS_EDTA_-CPL cryogel sorbents was obtained by the liquid displacement method [27]. Thus, a certain amount of dried microbeads (0.01 g) was immersed in a fixed volume of isopropanol (*V_1_*) for 5 min. As isopropanol is a non-solvent for the CS network, it enters only into the network pores but does not interact with its components. The sorbent porosity was calculated using Equation (3):

(3) $$P=\frac{V_{1}-V_{3}}{V_{2}-V_{3}}\times 100,$$

where *V_2_* is the total volume of isopropanol containing the immersed microbeads, and *V_3_* is the volume of isopropanol after the sample removal.

#### 4.2.4. Mean Pore Sizes Determination

The average pore sizes of all samples were obtained by Image J 1.48 v analyzing software [27]. In this regard, we measured at least 20 pores (voids) on each of three independent SEM micrographs taken for every sample.

#### 4.2.5. Water Uptake

The water uptake was evaluated by immersing a certain amount of dried microbeads (0.01 g) in 10 mL MilliQ water over a time span of 60 min. The swollen microbeads were weighed after wiping the excess of MilliQ water using a filter paper. The water uptake (g/g) was calculated as [41]:

(4) $$Water\;uptake=\frac{W_{w}-W_{d}}{W_{d}},$$

where *W_w_* and *W_d_* are the weights of swollen and freeze-dried microbeads, respectively.

#### 4.2.6. BET Surface Area

The BET surface area was estimated from dynamic water vapor sorption isotherms considering a relative humidity up to 40%. The experimental data were recorded at 25 °C using an IGAsorp gravimetric analyzer (Hiden Analytical, Warrington, UK), following the protocol previously applied for xanthan-based cryogels [42].

#### 4.2.7. Fixed-Bed Column Studies

Continuous fixed-bed sorption studies were performed using a multicomponent equimolar solution of Pb^2+^, Zn^2+^, Ni^2+^, Cd^2+^, and Co^2+^ (0.5 mmol/L of each metal ion) at pH 4.5 to investigate the efficacy of CS-CPL and CS_EDTA_-CPL beads in deep-cleaning of wastewaters in dynamic competitive conditions. The beads were packed (bed height = 5 cm) into a glass-column with the internal diameter of 1.45 cm that was connected to a Lambda Hiflow peristaltic pump (Lambda Laboratory Instruments, Brno, Czech Republic) at the top end to ensure a 1 mL/min constant flow rate of the multicomponent solution. Before passing the solution of heavy metal ions, the beads were equilibrated at pH 4.5 by passing an aqueous solution with the same pH through the column.

The effluent was automatically sampled in fractions of 10 mL using a Lambda OMNICOLL Fraction Collector (Lambda Laboratory Instruments, Brno, Czech Republic). The desorption of heavy metal ions was also studied in dynamic conditions by passing a 0.1 M HCl aqueous solution through the column at a flow rate of 1 mL/min.

The heavy metal ion concentrations in the collected samples were determined by Flame Atomic Absorption Spectrometry (FAAS) using a high-resolution ContrAA 300 Analytik Jena spectrometer (Analytik Jena, Jena, Germany) equipped with a Xenon lamp as a continuum radiation source. Measurements of each analyte were carried out in triplicate. The residual concentration of each metal ion was determined at the characteristic maximum wavelengths of 213.8 nm for Zn^2+^ ions, 217.0 nm for Pb^2+^, 228.8 nm for Cd^2+^, 232.0 nm for Ni^2+^ ions, and 240.7 nm for Co^2+^.

## Figures and Tables

**Table 1:** EDX elemental atomic composition of CS-CPL and CS_EDTA_-CPL microbeads surface.

Sample	EDX Elements Atomic Ratio, %							
	C	N	O	Al	Si	Na	K	Ca
CS-CPL	44.03	8.93	40.00	0.50	2.13	0.27	0.10	3.13
CS_EDTA_-CPL	47.97	9.90	35.50	0.50	2.00	0.27	0.17	2.83

**Table 2:** Some features of the CS-CPL and CS_EDTA_-CPL composite sorbents.

Sample Code	CPL, wt.%	Porosity, ^1^ %	Mean Pore Sizes, ^2^ μm	Water Uptake, ^3^ g/g	BET_H2O_ Data ^4^	
					Area, m^2^/g	Monolayer, g/g
CS-CPL	20	78.19 ± 2.04	21.49 ± 5.27	30.27 ± 3.48	431.16 ± 29.31	0.123 ± 0.084
CS_EDTA_-CPL	20	86.57 ± 1.31	30.16 ± 6.18	45.04 ± 4.82	450.34 ± 30.66	0.128 ± 0.067

^1^ Porosity was determined by liquid displacement method [27]; ^2^ mean pore sizes were evaluated from SEM micrographs using Image J 1.48v analyzing software; ^3^ water uptake was calculated with Equation (4) from Section 4. Materials and Methods; ^4^ BET_H2O_ surface area of composite sorbents was evaluated based on dynamic water vapor sorption isotherms considering a relative humidity up to 40%.

**Table 3:** Fitting parameters of Thomas and Yoon–Nelson models for the fixed-bed column experiments.

Sample	m ^1^ (mg)	Thomas			Yoon–Nelson		
		k_TH_ (L/min∙mg)	q_0_ (mg/g)	R^2^	k_YN_ (mL/min·mg)	τ (min)	R^2^
CS-CPL	109.9	5.38 × 10^−4^	68.72	0.985	0.133	30.64	0.985
CS_EDTA_-CPL	205.8	4.85 × 10^−5^	145.55	0.862	0.012	121.51	0.862

^1^ Sorbent mass loaded in the columns.

**Table 4:** Conformers of EDTA.

Conformer	Relative Energy (kcal/mole)	Spatial Structures ^1^	Dipole Moment (Debye)
EDTA-1	0.00	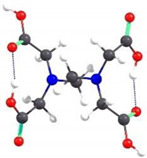	0.00
EDTA-2	6.44	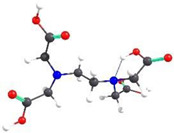	6.32
EDTA-3	6.72	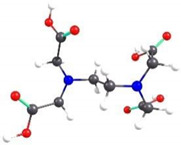	2.20
EDTA-4	8.88	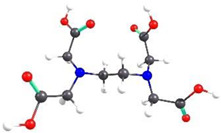	1.84
EDTA-5	16.09	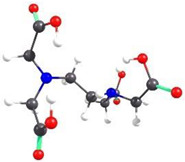	4.91

^1^ Legend colors: grey-C, red-O, blue-N, white-H.

**Table 5:** Conformers of DTPA.

Conformer	Relative Energy (kcal/mole)	Spatial Structures ^1^	Dipole Moment (Debye)
DTPA-1	0.00	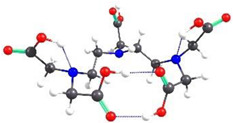	7.64
DTPA-2	1.88	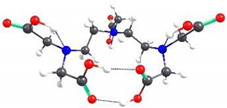	5.45
DTPA-3	1.92	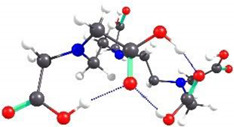	3.36
DTPA-4	5.98	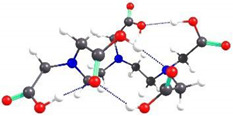	5.63
DTPA-5	6.44	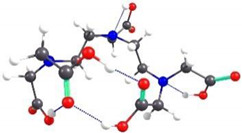	6.59

^1^ Legend colors: grey-C, red-O, blue-N, white-H.

## Data Availability

Not applicable.

## Supplementary Materials

The following supporting information can be downloaded at: https://www.mdpi.com/article/10.3390/gels8040221/s1, Table S1. Parameters corresponding to Thomas and Yoon–Nelson models fitted for each HMI on CS_EDTA_-CPL column. Table S2 [43,44,45,46,47,48,49]. Sorption performance of various sorbents reported in the literature compared with that of our CS_EDTA_-CPL sorbents. Figure S1. FT-IR spectra of CS-CPL composites before and after interaction with HMIs. Figure S2. FT-IR spectra of CS_EDTA_-CPL composites before and after interaction with HMIs.
