# Supplementary material for: Aminopolycarboxylic Acids-Functionalized Chitosan-Based Composite Cryogels as Valuable Heavy Metal Ions Sorbents: Fixed-Bed Column Studies and Theoretical Analysis
Source: Gels. 2022 Apr 5;8(4):221. doi: 10.3390/gels8040221 (PMC9030056; doi:10.3390/gels8040221)
Supplement: Supplementary file 1 [file gels-08-00221-s001.zip › gels-1655239-supplementary.pdf]

gels

MDPI

Article

Aminopolycarboxylic Acids-Functionalized Chitosan-Based Composite Cryogels as Valuable Heavy Metal Ions Sorbents: Fixed-Bed Column Studies and Theoretical Analysis

Maria Valentina Dinu [1,*], Ionel Humelnicu [2], Claudiu Augustin Ghiorghita [1] and Doina Humelnicu [2]

[1] "Mihai Dima" Department of Functional Polymers, "Petru Poni" Institute of Macromolecular Chemistry, Grigore Ghica Voda Alley 41A, 700487 Iasi, Romania; claudiu.ghiorghita@icmpp.ro
[2] Faculty of Chemistry, "Al. I. Cuza" University of Iasi, Carol I Bd. 11, 700506 Iasi, Romania; ionel@uaic.ro (I.H.); doinah@uaic.ro (D.H.)
* Correspondence: vdinu@icmpp.ro

Supporting Information

Table S1. Parameters corresponding to Thomas and Yoon-Nelson models fitted for each HMI on CS_{EDTA}-CPL column.

HMI	Thomas			Yoon-Nelson		
	k_{TH} (L/min·mg)	q_0 (mg/g)	R^2	k_{YN} (mL/min·mg)	τ (min)	R^2
Co(II)	$1.55·10^{-4}$	79.57	0.9365	$3.83·10^{-2}$	66.43	0.9365
Zn(II)	$6.29·10^{-5}$	130.03	0.8888	$1.55·10^{-2}$	108.56	0.8888
Cd(II)	$4.10·10^{-5}$	158.72	0.8173	$1.01·10^{-2}$	132.51	0.8173
Pb(II)	$3.31·10^{-5}$	166.78	0.7950	$8.16·10^{-3}$	139.24	0.7950
Ni(II)	$3.52·10^{-5}$	249.53	0.8594	$8.68·10^{-3}$	208.34	0.8594

Table S2. Sorption performance of various sorbents reported in the literature compared with that of our CS_{EDTA}-CPL sorbents.

Sorbent	Metal ion	pH	Sorption operation conditions	q_0, mg/g	Refs.
Zeolite-supported nanoscale zero-valent iron	Pb(II)<br>Cd(II)	6	batch	85.37<br>48.63	[1]
Iron coating zeolite	Pb(II)<br>Cd(II)<br>Zn(II)	6.5	batch	6.54<br>4.20<br>3.72	[2]
Iron coating zeolite	Pb(II)<br>Cd(II)<br>Zn(II)	6.5	column	1.03<br>0.93<br>0.83	[2]
Lemon peel-based biomaterial	Cd(II)<br>Co(II)<br>Ni(II)<br>Pb(II)	5	batch	7.34<br>5.63<br>5.73<br>8.17	[3]
PVP hydrogel	Ni(II)<br>Zn(II)<br>Cd(II)	8	batch	7.52<br>26.78<br>4.35	[4]
P(VP-co-MA) hydrogel	Ni(II)<br>Zn(II)<br>Cd(II)	8	batch	60.15<br>48.92<br>29.82	[4]
FAU-type zeolite	Pb(II)<br>Cd(II)<br>Zn(II)<br>Co(II)	Not specified	batch	109.9<br>53.5<br>36.8<br>12.2	[6]
Mesoporous magnetite nanoparticles	Pb(II)<br>Cd(II)<br>Ni(II)	5.5	batch	85<br>79<br>66	[7]
Copper-imprinted CS-based cryogel	Ni(II)<br>Zn(II)	4.5<br>5	batch	77.67<br>84.14	8
CS_{EDTA}-CPL	Co(II)<br>Zn(II)<br>Cd(II)<br>Pb(II)<br>Ni(II)	4.5	column	79.57<br>130.03<br>158.72<br>166.78<br>249.53	this study

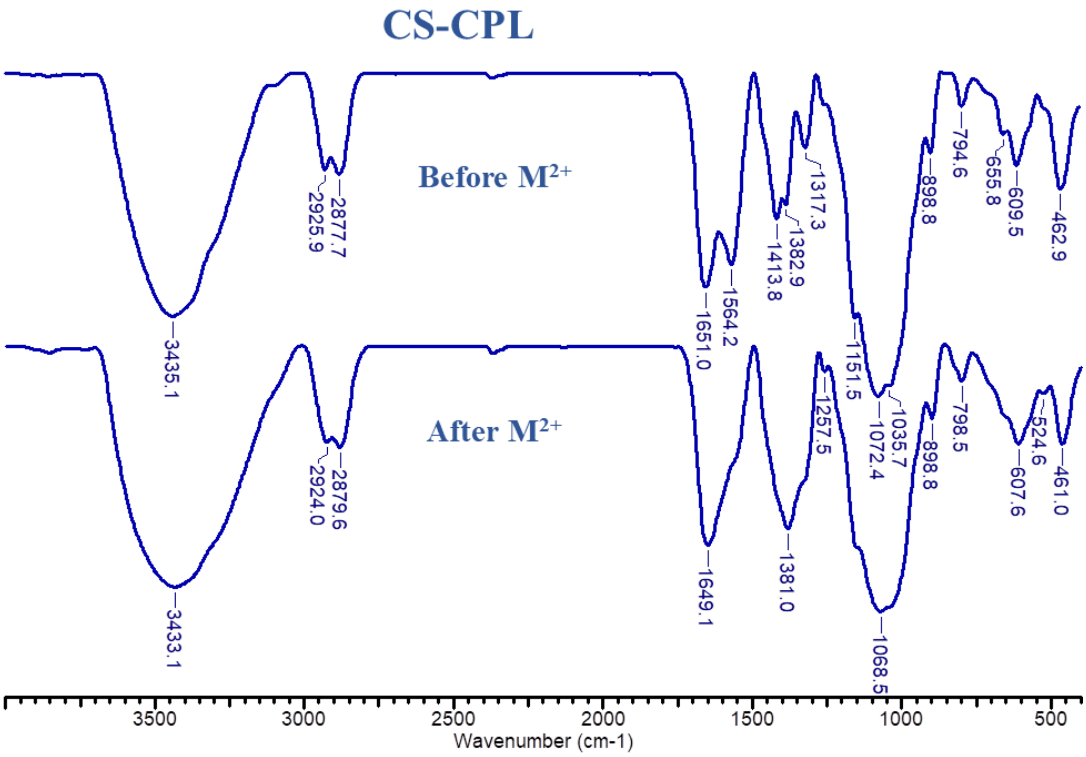

Figure S1. FT-IR spectra of CS-CPL composites before and after interaction with HMIs.

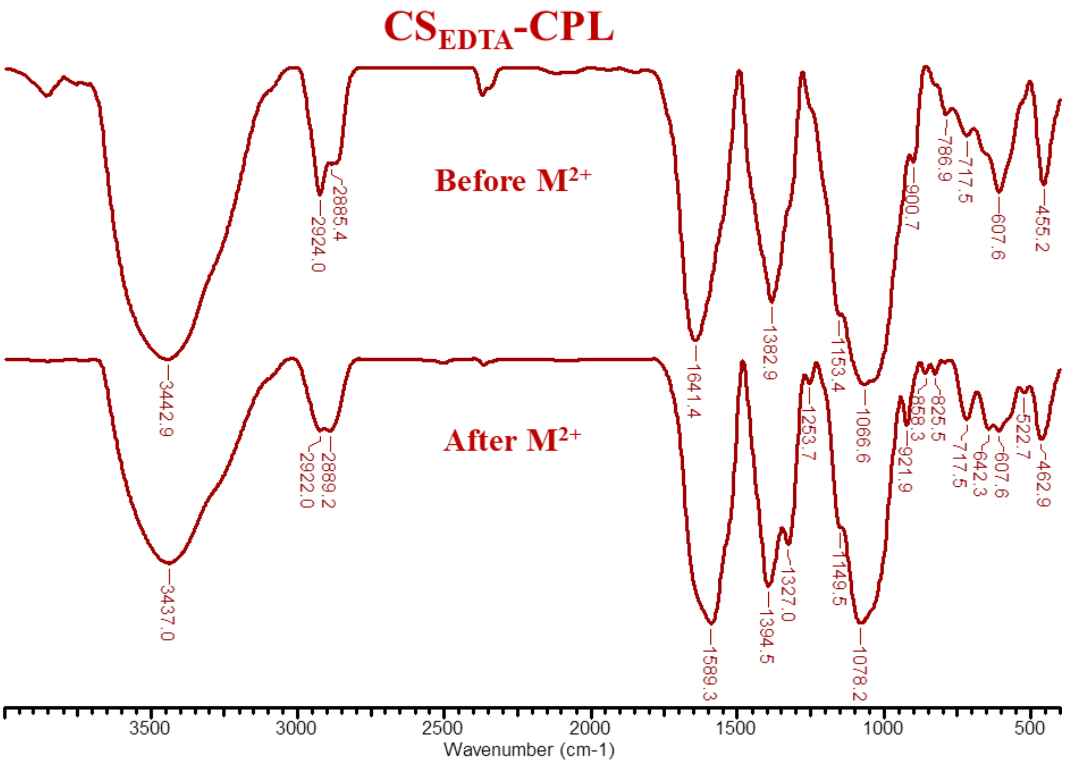

Figure S2. FT-IR spectra of CS_{EDTA}-CPL composites before and after interaction with HMIs.

References

1. Li, Z., Wang, L., Meng, J., Liu, X., Xu, J., Wang, F., Brooks, P., Zeolite-supported nanoscale zero-valent iron: New findings on simultaneous adsorption of Cd(II), Pb(II), and As(III) in aqueous solution and soil. *J. Hazard. Mater.* **2018**, *344*, 1–11.
2. Nguyen, T. C., Loganathan, P., Nguyen, T. V., Vigneswaran, S., Kandasamy, J., Naidu, R., Simultaneous adsorption of Cd, Cr, Cu, Pb, and Zn by an iron-coated Australian zeolite in batch and fixed-bed column studies. *Chem. Eng. J.*, **2015**, *270*, 393–404.
3. Šabanović, E., Memić, M., Sulejmanović, J., Selović, A., Simultaneous adsorption of heavy metals from water by novel lemon-peel based biomaterial, *Polish J. Chem. Technol.*, **2020**, *22*, 46-53.
4. Kemik, Ö.F., Ngwabebhoh, F.A., Yildiz, U., A response surface modelling study for sorption of Cu^{2+}, Ni^{2+}, Zn^{2+} and Cd^{2+} using chemically modified poly(vinylpyrrolidone) and poly(vinylpyrrolidone-co-methylacrylate) hydrogels. *Adsorp. Sci. Technol.*, **2017**, *3*, 263-283.
5. Joseph, I.V., Tosheva, L., Doyle, A. M., Simultaneous removal of Cd(II), Co(II), Cu(II), Pb(II), and Zn(II) ions from aqueous solutions via adsorption on FAU-type zeolites prepared from coal fly ash. *J. Environm. Chem. Eng.*, **2020**, *8*, 103895.
6. Fato, P.F., Li, D.W., Zhao, L.J., Qiu, K., Long, Y.T., Simultaneous Removal of Multiple Heavy Metal Ions from River Water Using Ultrafine Mesoporous Magnetite Nanoparticles, *ACS Omega*, **2019**, *4*, 7543-7549.
7. Humelnicu, D., Lazar, M.M., Ignat, M., Dinu, I.A., Dragan, E.S., Dinu, M.V., Removal of heavy metal ions from multi-component aqueous solutions by eco-friendly and low-cost composite sorbents with anisotropic pores, *J. Hazard. Mater.*, **2020**, *381*, 120980.
